# A systematic review of outcomes measured in interventional trials in people with diabetic sensorimotor polyneuropathy

**DOI:** 10.1111/dme.70134

**Published:** 2025-09-12

**Authors:** Galvin Chiam, Sasha Smith, Tony Tu, Amaan Din, Pasha Normahani, Alun Davies

**Affiliations:** ^1^ Section of Vascular Surgery, Department of Surgery and Cancer, Charing Cross Hospital Imperial College London London UK; ^2^ Imperial Vascular Unit, Charing Cross Hospital Imperial College Healthcare NHS Trust London UK

**Keywords:** diabetes mellitus, diabetic neuropathy, diabetic sensorimotor polyneuropathy, nervous system outcomes, outcome measure, pain

## Abstract

**Aims:**

Diabetic sensorimotor polyneuropathy (DSPN) is the most common chronic complication of diabetes. Heterogeneity in outcome measures across DSPN trials may have hindered the development of novel therapies. No core outcome set (COS) exists to standardise DSPN trial outcomes. This systematic review aims to identify and synthesise outcomes reported in DSPN interventional trials.

**Methods:**

The protocol was pre‐registered on PROSPERO (CRD42023408403) and reported in line with Preferred Reporting Items for Systematic Reviews and Meta‐Analysis (PRISMA) guidelines. Prospective DSPN interventional trials since 2018 were searched with a predefined strategy, and primary and secondary verbatim outcomes were extracted, merged and organised using a taxonomy recommended by Core Outcome Measures in Effectiveness Trials (COMET). Outcome measuring tools were summarised descriptively.

**Results:**

Of the 4851 abstracts screened, 184 were eligible (protocols, *n* = 24; ongoing trials, *n* = 48 completed trials without published results, *n* = 11; published trials with results, *n* = 101). Pain was the most common primary (*n* = 127) and secondary (*n* = 64) unique outcome. By taxonomy, nervous system outcomes were the most common primary (*n* = 174) and secondary (*n* = 89) measure. The most common measuring tools were the visual analogue scale (*n* = 37), numerical rating scale (*n* = 37) and nerve conduction study (*n* = 34). Over 30 distinct measuring tools were utilised to measure nervous system outcomes.

**Conclusions:**

Despite consistent outcome reporting, variability in measuring tools highlights the need for a COS with standardised tools. Patient‐reported outcomes were more common than assessor‐reported outcomes; however, using both may reduce response variability and bias. These findings will inform a future Delphi process to develop a COS for DSPN.

**What's new?**

- This systematic review is the first to utilise a taxonomy recommended by the Core Outcome Measures in Effectiveness Trials (COMET) initiative to quantitatively investigate the outcome reporting and measuring tools of interventional diabetic sensorimotor polyneuropathy trials.
- Pain was the most common primary and secondary outcome measure, with the numerical rating scale and visual analogue scale being the most common primary measuring tools.
- The variability in measuring tools in existing literature highlights the need for the development of a core outcome set with standardised tools for interventional diabetic sensorimotor polyneuropathy trials.

## 1 INTRODUCTION

Diabetes is an increasingly prevalent chronic metabolic condition.^1^ In 2021, the global prevalence was 537 million, with 6.7 million associated deaths.^2^ This is expected to increase to 643 million by 2030,^1^ comprising mostly of type 2 diabetes (T2DM) cases.^1^ A major complication of diabetes is diabetic sensorimotor polyneuropathy (DSPN), characterised by lower limb peripheral nerve damage that spreads proximally.^3^ A landmark multi‐centre cross sectional study in the United Kingdom (UK) found that 28.5% of people with diabetes seen in secondary care were affected by DSPN.^4^ Additionally, a recent large‐scale community‐based study in the UK, involving more than 15,000 participants with diabetes, reported that 34% experienced DSPN‐related pain, known as ‘painful DSPN’.^5^ The study also found that the adjusted risk of painful DSPN amongst individuals with T2DM was double that of individuals with type 1 diabetes (T1DM), with an odds ratio of 2.1.^5^ The lifetime prevalence of DSPN in people with diabetes is estimated to exceed 50%,^6^ often leading to further complications such as diabetic foot ulcers and Charcot foot, which are both independently associated with increased mortality.^3^ Given the burgeoning rate of diabetes worldwide, effective prevention and management of DSPN are becoming ever so crucial.

DSPN is a length‐dependent polyneuropathy, initially affecting the longest nerves and presenting with a ‘stocking and glove’ distribution.^3^ The clinical manifestations result from the loss of both small and large nerve fibres. The symptoms of the small fibre loss include neuropathic pain, hyperalgesia and allodynia, while numbness and dysesthesia fall under large fibre loss.^3^ People with DSPN may exhibit mixed symptoms, or even appear asymptomatic,^3^ making diagnosis difficult. A systematic review investigating the association between DSPN and anxiety, depression and sleep disorders revealed that the prevalence of sleep disorders in people with painful DSPN was more than 40%,^7^ and anxiety and depression coexist in up to 30% of people with painful DSPN.^7^ It is evident that DSPN significantly impacts both the quality of life of those affected,^8^ manifesting in complex, multi‐faceted ways that compromise both physical and psychosocial function.

DSPN also carries a heavy socioeconomic burden. In 2011, the healthcare costs of DSPN in the UK were more than £300 million, with projections of an increase to over £500 million by 2035.^9^ Additionally, managing further complications such as diabetic foot ulcers and amputations was estimated to cost almost £1 billion in 2011, with a two‐fold increase expected by 2035.^9^ Consequently, DSPN presents not only a clinical challenge but also a significant financial strain on the National Health Service (NHS) in the UK.

Despite such a high prevalence, the exact pathophysiology of DSPN is yet to be determined. Studies have shown that one process driving nerve dysfunction is hyperglycaemia‐induced cellular injury, which is further exacerbated by the presence of comorbidities such as obesity, insulin resistance and dyslipidaemia.^1^ Chronic hyperglycaemia raises the intracellular levels of reactive oxidative species (ROS) through enzymatic reduction of glucose and the extracellular level of advanced glycated end products (AGEs) through non‐enzymatic conversion of proteins, lipids and nucleic acids in glucose‐sensitive axons and Schwann cells. These mechanisms activate downstream receptors, contributing to oxidative stress and inflammation. The resulting damage includes demyelination, impaired axonal function and reduced blood flow to peripheral nerves, compromising nerve conduction.^10^, ^11^

Currently, there are no approved treatments to reverse DSPN. However, there are prevention measures recommended by numerous healthcare guidelines to slow progression. The National Institute for Health and Care Excellence (NICE) in the UK recommends improving glycaemic control as a first‐line measure to slow the development of DSPN,^12^ and advanced glucose monitoring devices have made improving glycaemic control more accessible and personalised for people with diabetes. Additionally, lifestyle modifications such as dietary changes and increasing physical activity reduce the risk of DSPN and improve patients' overall cardiometabolic profile.^12^

Pharmacological treatments largely focus on managing neuropathic pain in people with painful DSPN. The most ubiquitous drugs administered are anticonvulsants such as pregabalin or gabapentin, serotonin and norepinephrine re‐uptake inhibitors such as duloxetine and tricyclic antidepressants such as amitriptyline.^13^ However, side effects of these drugs are common. For example, amitriptyline can cause urinary retention and postural hypotension, which makes prescription for elderly patients more challenging.^14^ Non‐pharmacological treatments include neuromodulation devices such as spinal cord stimulation, which administers electrical impulses through the epidural space during neuropathic pain episodes.^15^ Although the exact mechanism of action for spinal cord stimulation is not fully understood, it is approved by the Medicines and Healthcare products Regulatory Agency (MHRA) in the UK and the Food and Drug Administration (FDA) in the United States of America (USA) for refractory neuropathic pain.^16^

A myriad of novel interventions, both pharmacological and non‐pharmacological, has been developed and tested in clinical trials. However, evaluating the evidence for these interventions is challenging due to the high heterogeneity of outcomes across trials. For example, a crucial limitation identified by Waldfogel et al.^17^ and Nesbit et al.^18^ in their systematic reviews of treatments for DSPN was the presence of incomplete and inconsistent reporting, where different trials measure different outcomes, hindering comparisons and pooling of data.

A Core Outcome Set (COS) is an agreed set of standardised reporting outcomes. The development of a COS has been initiated for interventional trials in T2DM and diabetic foot ulcers because previous systematic reviews in these areas have identified a lack of consistent outcome reporting.^19^, ^20^ In particular, a review of T2DM interventional trials found no single outcome or outcome domain measured in all included studies. Establishing a COS for DSPN interventional trials may improve outcome reporting, enabling comparisons and pooling of data, and promote the measurement of outcomes that are pertinent and meaningful to patients, clinicians and other stakeholders involved in disease management. In turn, this may prevent research wastage and contribute to the development of high‐quality evidence that can support the development of treatments, healthcare guidelines and policies.^21^

As recommended by the Core Outcome Measures in Effectiveness Trials (COMET) initiative,^21^ the first step in developing a COS is to determine which outcomes should be measured. The aim of this systematic review was to identify and synthesise the outcomes reported in recent interventional trials in people with DSPN and to highlight gaps in the existing literature, in order to provide a foundation for COS development. We hypothesised that there would be considerable variability in outcomes measured across interventional DSPN trials.

## 2 METHODS

This systematic review was reported in line with the Preferred Reporting Items for Systematic Reviews and Meta‐Analysis (PRISMA) guidelines.^22^ The protocol was pre‐registered on the PROSPERO database (CRD42023408403). This review forms part of the DEveloping a Core Outcome set for Diabetic nEuropathy clinical trials (DECODE) project registered on the COMET Database.^23^

### 2.1 Eligibility criteria

Prospective clinical trials assessing treatments in adults with DSPN were included in this review. The full eligibility criteria are displayed in Table 1.

**TABLE 1:** Inclusion and exclusion criteria used when assessing interventional diabetic sensorimotor polyneuropathy (DSPN) trials generated from the literature search for eligibility.

Inclusion criteria	Exclusion criteria
Prospective clinical studies	Retrospective studies
Clinical trials assessing an intervention to treat DSPN	Observational studies
Studies with participants aged ≥18 years old	Systematic reviews, narrative reviews, expert opinions and editorials
Studies with ≥50 participants	Abstracts and conference proceedings
	Studies in animals
	Studies not available in English
	Studies published before 1 January 2018
	Studies aimed exclusively at assessing cost‐effectiveness
	Studies aimed exclusively at assessing interventions for the prevention of DSPN
	Studies aimed exclusively at assessing diagnostic tools
	Studies aimed exclusively at assessing interventions for complications of DSPN (e.g. ulceration, Charcot foot)
	Studies aimed exclusively at assessing interventions for other types of neuropathies

Clinical trials at four different stages were included (protocols, ongoing trials, completed trials without published results and published trials with results). Ongoing trials were only included if the outcomes were prespecified in a protocol. In the scenario where a study had both a protocol and a results paper, only the results paper was included. Trials were only included if at least 50 participants remained in follow‐up and were included in the analysis. For trials with fewer than 50 participants but had a protocol with a target sample size of more than 50, only the protocol was included.

### 2.2 Search strategies

Ovid MEDLINE® and Embase databases, as well as the Cochrane Central Register of Controlled Trials (CENTRAL) database were searched on 8 June 2023. The literature search was limited to articles published since 1 January 2018, to ensure a comprehensive yet manageable selection of relevant studies, offering an overview of outcome reporting in the most recent DSPN trials. The full search strategy is available in Appendix S1.

Systematic reviews identified in the search were ineligible, but manual citation searching was performed on these articles to identify additional trials excluded from the registry and database search.

### 2.3 Article screening

The article data were exported to Covidence (Melbourne, Australia). The titles and abstracts were screened by two independent reviewers based on the eligibility criteria. In the event of conflicting decisions between the two reviewers, the inclusion of these trials was decided by a third, independent reviewer. Upon passing the first round of screening, full‐text versions of included trials were screened by two reviewers for eligibility, and conflicts were decided by a third, independent reviewer.

### 2.4 Data extraction and analysis

Data extraction was performed using a Qualtrics (Seattle, United States of America) form (Appendix S2). Qualtrics was used firstly because of its in‐built validation rules and logic to prevent entry errors and ensure all necessary fields were completed. Secondly, all fields were centralised and accessible to the reviewers, facilitating simultaneous data extraction. Lastly, data were easily exportable and visualised using the built‐in software. Pilot data extraction was performed on 10 trials to ensure the Qualtrics form captured the required data. Data extraction was primarily completed by one reviewer. However, given the subjective nature of the process, the verbatim extraction of primary and secondary outcomes and classification into unique outcomes was completed independently by two reviewers. Any discrepancies in the classification of unique outcomes were then adjudicated by a third senior reviewer (Appendix S3). To ensure no landmark studies were missed that could have impacted our results, outcome saturation was assessed in reverse chronological order of publication year in order to ensure no new unique outcomes were extracted.

The trials were stratified based on trial stage (i.e. protocol, ongoing trial, completed trial without published results and published trial with results), and the following study characteristics were extracted: study design, number of centres, location, intervention type, sample size, participant demographics, source of funding, number of follow‐up visits and total follow‐up duration. Intervention type was categorised into pharmacological (i.e. drugs and nutraceuticals) and non‐pharmacological treatments. Participant demographics referred to whether the trial included participants with T1DM, T2DM or both, and whether all participants had DSPN.

A verbatim description of primary and secondary outcomes was extracted from the text, as well as the relevant measuring tools used. The prespecified and actual outcomes measured were both extracted. These outcomes and measuring tools were further categorised into self reported and assessor‐reported. Self reported was defined as outcomes or measuring tools which were reported by participants, while assessor‐reported was defined as outcomes or measuring tools which were observed or measured by assessors. For example, the EuroQoL‐5D measures quality of life via a participant‐completed questionnaire.^24^ Conversely, nerve conduction studies (NCS) require trained electromyographers to assess nerve function.^25^ Additionally, unique primary and secondary outcomes were separately classified into domains using the outcome taxonomy produced by Dodd et al.,^26^ as recommended by COMET.^21^

To homogenise data, the primary outcome duration and total follow‐up duration were converted to weeks. For example, ‘6 months’ was converted to ‘26 weeks’. To prevent duplication of unique outcomes, different methods of measuring the same outcome was classified as one outcome. For example, if the outcome ‘pain’ was measured numerous times using different scoring systems, it was only considered as one unique outcome. Temporality of an outcome was not considered in defining if an outcome was unique. For instance, ‘daily average pain’ and ‘weekly average pain’ were both classified under the outcome ‘pain’. Outcomes with similar meanings were merged, using the framework postulated by Young et al.,^27^ which suggests that outcomes with different words, phrasing or spelling that refer to the same concept should be grouped as one outcome. An example includes grouping ‘fasting blood sugar levels’ and ‘fasting blood glucose levels’ as one outcome. Another would be categorising ‘unsteadiness’ and ‘ataxia’ as the same outcome.

Descriptive statistics (frequencies, percentages) were used to analyse data extracted from the interventional DSPN trials. A meta‐analysis was not performed because the aim of the systematic review was to identify and synthesise outcomes reported in DSPN interventional trials, not to assess treatment effects. The findings were presented in the form of frequency tables to display study characteristics, outcomes reported and measuring tools used. The outcomes by taxonomy and outcome/study durations were presented using bar graphs. A frequency table was dedicated to the nerves and parameters measured as part of NCS, which remains the gold standard for diagnosing and assessing DSPN.^3^ Outcomes by taxonomy and follow‐up duration were represented by bar graphs.

## 3 RESULTS

**FIGURE 1:**
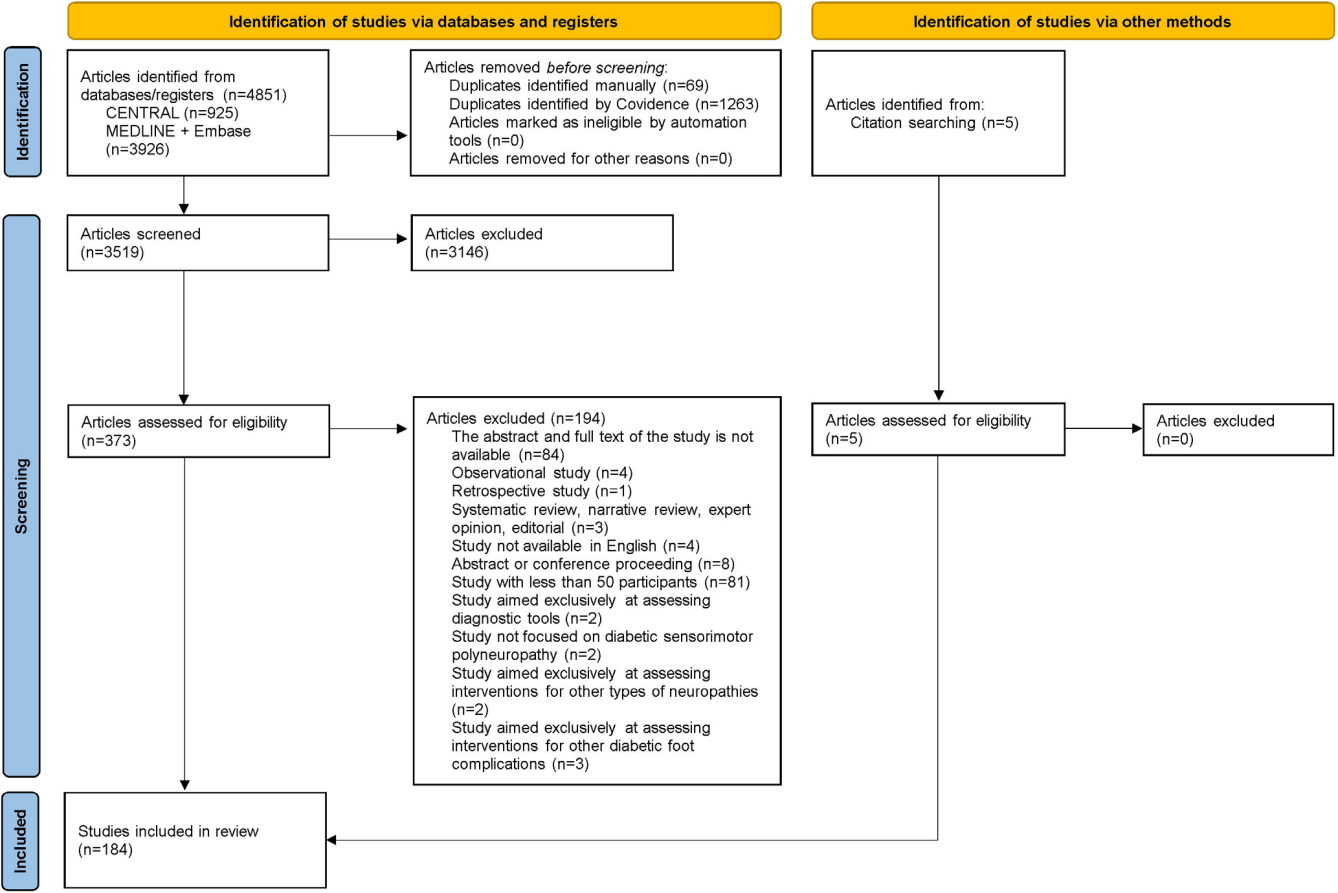
Preferred Reporting Items for Systematic Reviews and Meta‐Analysis (PRISMA)^22^ flow diagram of interventional DSPN trials.

**FIGURE 2:**
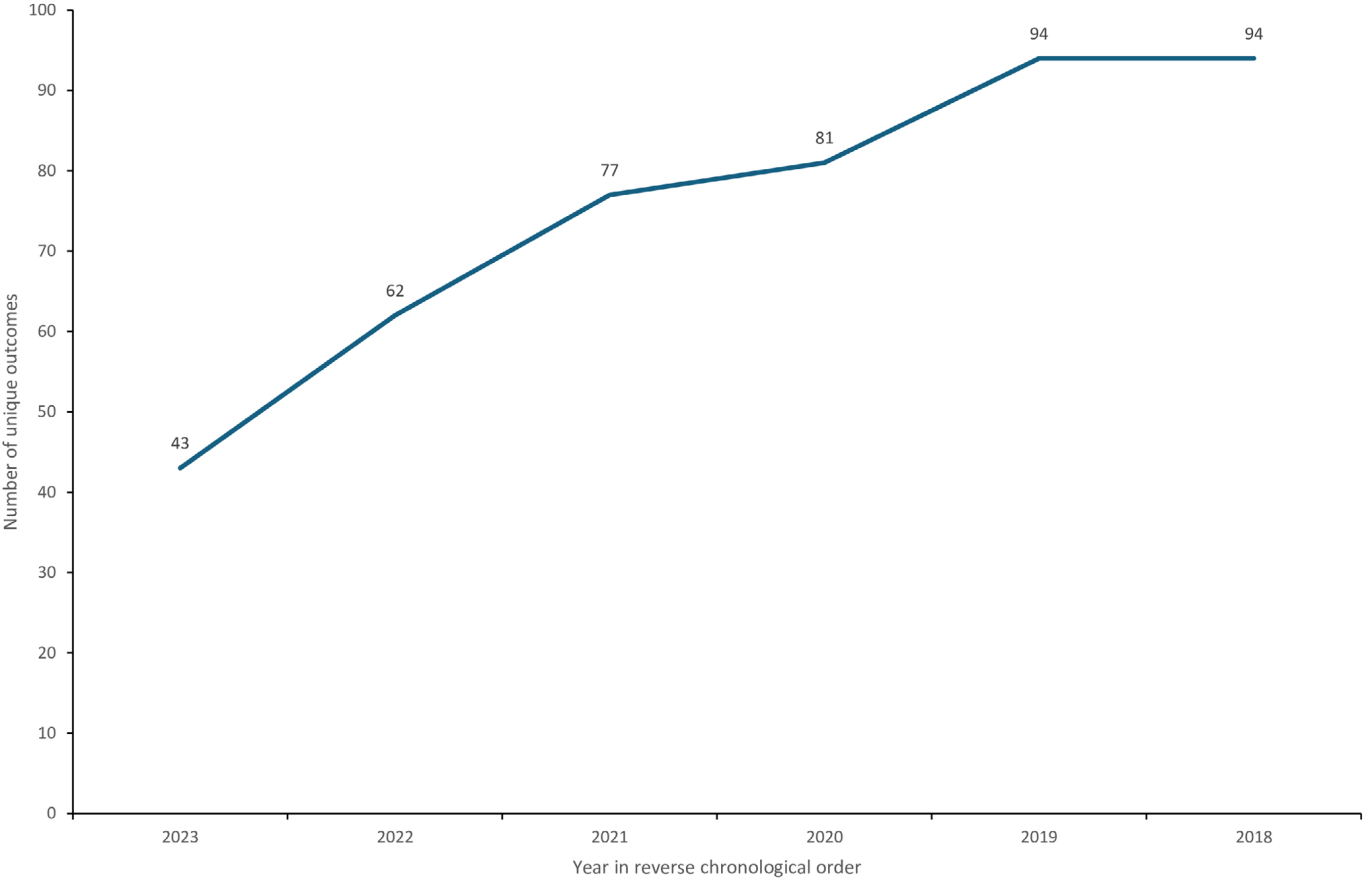
Number of unique outcomes indexed in reverse chronological order from 2023 to 2018.

The database searches identified 4851 articles. This included 107 systematic reviews that were manually citation‐searched to identify five eligible trials. After the removal of 1332 duplicates, 3519 articles had their titles and abstracts screened. After the exclusion of 3146 articles, the full texts of 373 articles were then screened for eligibility. After the exclusion of 194 articles, 179 trials were eligible. The majority of excluded studies following full‐text review were due to a small sample size (*n* = 79) or an unretrievable full text (*n* = 84). Studies were excluded as unretrievable full text primarily when they were listed in trial registries providing only summary information, or when published in journals inaccessible through institutional library or inter‐library loan services. Considering the further five eligible trials identified from the manual citation search, a total of 184 trials were included in this review (Figure 1). There were a total of 94 unique outcomes, and outcome saturation in reverse chronological order was achieved by 2019 (Figure 2). Therefore, an extended search for studies was not required.

### 3.1 Study characteristics

The 184 included trials comprised 24 protocols, 48 ongoing trials, 11 completed trials without published results and 101 published trials with results (Table 2). The majority of the trials included were randomised controlled trials (*n* = 166) consisting solely of people with DSPN (*n* = 162). About two‐thirds of the trials operated with a single centre (*n* = 127), and most of the trials were based in Asia (*n* = 130). About half of the trials stated that public funding was used (*n* = 93), while about a quarter were industry‐funded (*n* = 48), and the rest did not mention the source of financial support (*n* = 43). Most of the trials prespecified the outcomes measured and timepoints in which they would be measured (*n* = 160), and trials most commonly had only one follow‐up visit (*n* = 84).

**TABLE 2:** Characteristics of 184 data extracted trials, stratified by trial stage.

Parameter	Protocol (*n* = 24)	Ongoing trial (*n* = 48)	Completed trial without published results (*n* = 11)	Published trial with results (*n* = 101)
Study design				
RCT (*n* = 166)	24	40	10	92
Non‐RCT (*n* = 18)	0	8	1	9
Number of centres				
Single centre (*n* = 127)	11	43	6	67
Multi‐centre (*n* = 57)	13	5	5	34
Geographical region				
North America (*n* = 11)	1	1	0	9
South America (*n* = 3)	3	0	0	0
Europe (*n* = 28)	6	3	2	17
Africa (*n* = 6)	1	1	0	4
Asia (*n* = 130)	12	42	8	68
Australia (*n* = 3)	1	1	0	1
International (*n* = 3)	0	0	1	2
Intervention type				
Pharmacological (*n* = 110)	7	25	10	68
Non‐pharmacological (*n* = 70)	17	21	1	31
Both (*n* = 4)	0	2	0	2
Sample size				
50–100 (*n* = 103)	10	31	6	56
101–200 (*n* = 50)	5	14	2	29
>200 (*n* = 31)	9	3	3	16
Presence of DSPN				
All participants (*n* = 162)	20	46	9	87
Not all participants (*n* = 22)	4	2	2	14
Diabetes type				
T1DM (*n* = 0)	0	0	0	0
T2DM (*n* = 94)	9	27	4	54
Both (*n* = 37)	6	6	4	21
Not specified (*n* = 53)	9	15	3	26
Funding				
Public (*n* = 93)	18	37	4	34
Industry (*n* = 48)	5	8	7	28
Not specified (*n* = 43)	1	3	0	39
Prespecified outcome				
Outcome and timepoint (*n* = 160)	23	36	10	91
Outcome without timepoint (*n* = 24)	1	12	1	10
Not prespecified (*n* = 0)	0	0	0	0
Number of follow‐up visits				
1 (*n* = 84)	2	35	5	42
2 (*n* = 32)	6	8	0	18
3 (*n* = 24)	5	1	1	17
4 (*n* = 19)	2	1	3	13
5 (*n* = 10)	2	2	0	6
6 (*n* = 4)	2	0	0	2
7 (*n* = 3)	1	0	1	1
8 (*n* = 3)	2	0	1	0
9 (*n* = 3)	2	0	0	1
≥10 (*n* = 2)	0	1	0	1

Abbreviations: DSPN, diabetic sensorimotor polyneuropathy; non‐RCT, non‐randomised controlled trial; RCT, randomised controlled trial; T1DM, type 1 diabetes mellitus; T2DM, type 2 diabetes mellitus.

### 3.2 Unique outcomes

Table 3 displays the frequency of the 20 most common unique outcomes measured in the trials, after the merging of overlapping terminology using Young et al.'s^27^ published framework. The most frequent outcome was pain, with it being a primary outcome in over two‐thirds of interventional trials (*n* = 127), and a secondary outcome in about a third of trials (*n* = 64). Of the 184 studies, 155 included pain as a primary or secondary outcome. The most common assessor‐reported primary and secondary outcomes were nerve conduction velocity (*n* = 41) and adverse events (*n* = 48), respectively. Meanwhile, pain was the most common self reported primary (*n* = 127) and secondary outcome (*n* = 64).

**TABLE 3:** Frequency of the 20 most common unique primary and secondary outcomes across the 184 included trials, categorised by method of reporting.

Outcome	Primary *n* (%)	Secondary *n* (%)
Self reported		
Pain	127 (69.0)	64 (34.8)
Quality of life	17 (9.2)	41 (22.3)
DSPN‐related complaints	14 (7.6)	8 (4.3)
Daily functioning	7 (3.8)	16 (8.6)
Patient global impression of change	5 (2.7)	30 (16.3)
Anxiety and depression	5 (2.7)	22 (12.0)
Assessor‐reported		
Nerve conduction velocity	41 (22.3)	26 (14.1)
Vibration	39 (21.2)	19 (10.3)
Numbness	38 (20.7)	21 (11.4)
Reflexes	36 (20.0)	21 (11.4)
Temperature	36 (20.0)	21 (11.4)
Tingling	27 (14.7)	13 (11.4)
Ataxia	23 (12.5)	16 (8.7)
Light touch	21 (11.4)	11 (6.0)
Action potential amplitude	18 (9.8)	15 (8.2)
Blood glucose	15 (8.2)	25 (13.6)
Pinprick	15 (8.2)	9 (4.9)
Adverse events	14 (7.6)	48 (26.1)
Foot ulcers/appearance	13 (11.4)	9 (4.9)
Weakness	13 (11.4)	8 (4.3)

*Note*: *n* equates to the number of included trials, which measured the specified outcome, and the percentage reflects the proportion of the 184 included trials.

### 3.3 Nerve conduction studies

Table 4 displays the frequency of nerves and conduction parameters assessed through NCS, based on unique outcomes measured in the trials. For motor nerves, the most frequently assessed was the peroneal nerve for a primary outcome (*n* = 13) and the tibial nerve for secondary outcomes (*n* = 8). For sensory nerves, the sural nerve was the most frequently assessed for both primary (*n* = 13) and secondary (*n* = 10) outcomes. In terms of conduction parameters, the most common was nerve conduction velocity (primary *n* = 41; secondary *n* = 26), followed by action potential amplitude (primary *n* = 18; secondary *n* = 15).

**TABLE 4:** Frequency of nerves and conduction parameters assessed through nerve conduction studies, based on unique outcomes across the 184 included trials.

Nerve conduction studies	Primary *n* (%)	Secondary *n* (%)
Motor nerves		
Peroneal	13 (7.1)	7 (3.8)
Tibial	7 (3.8)	8 (4.3)
Median	7 (3.8)	4 (2.2)
Sensory nerves		
Sural	13 (7.1)	10 (5.4)
Peroneal	6 (3.3)	4 (2.2)
Median	5 (2.7)	5 (2.7)
Outcome parameter		
Nerve conduction velocity	41 (22.3)	26 (14.1)
Action potential amplitude	18 (9.8)	15 (8.2)
Onset latency	5 (2.7)	7 (3.8)

*Note*: *n* equates to the number of included trials which measured the specified nerve and parameter, and the percentage reflects the proportion of the 184 included trials.

### 3.4 Outcomes by taxonomy

**FIGURE 3:**
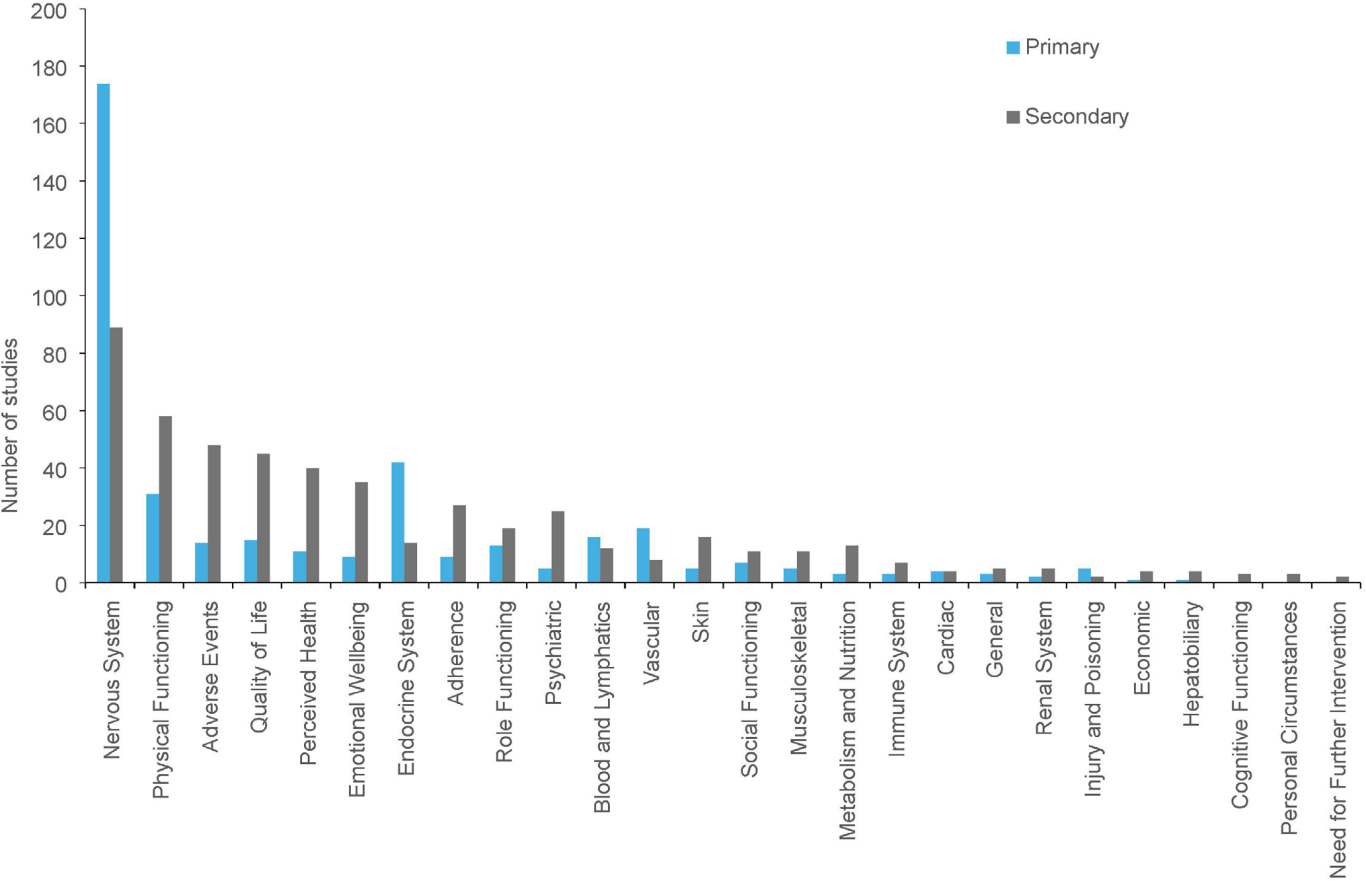
Frequency of primary and secondary outcomes across the studies by taxonomy published by Dodd et al.^26^

Figure 3 displays the frequency of outcomes measured in the trials according to the taxonomy published by Dodd et al.^26^ Nervous system outcomes, which can include symptoms such as pain or objective measurements such as nerve conduction velocity, were the most frequently measured, with almost every trial including this type as a primary outcome (*n* = 174) and almost half of trials including this type as a secondary outcome (*n* = 89). By taxonomy, the endocrine system was the second most common primary outcome (*n* = 42), while physical functioning was the second most common secondary outcome measured (*n* = 58). This type consisted of 22 unique outcomes ranging from sleep interference to walking speed. The least frequently measured outcomes were cognitive functioning (*n* = 3), personal circumstances (*n* = 3) and need for further intervention (*n* = 2), all of which were measured only as secondary outcomes.

### 3.5 Measuring tools

Across the 184 trials, primary and secondary outcomes were measured using a variety of tools. Table 5 displays the 20 most common outcome measuring tools used across the trials. All 20 of the measuring tools displayed measure an outcome which was amongst the five most common outcomes by taxonomy, namely nervous system, physical functioning, quality of life and perceived health outcomes (Figure 3). The most common measuring tool for pain as a primary outcome was the numerical rating scale (NRS) (*n* = 37) and visual analogue scale (VAS) (*n* = 37). The most common self reported measuring tool for secondary outcomes was the patient global impression of change (*n* = 31), followed by the NRS (*n* = 24). The most common assessor‐reported tool for both primary and secondary outcomes was NCS (primary *n* = 34; secondary *n* = 18). The outcomes by taxonomy with the greatest number of distinct scoring methods were physical functioning (*n* = 35), followed by nervous system (*n* = 32) and quality of life (*n* = 12). Of the 35 measuring tools used to assess physical functioning, 13 evaluate sleep quality, while 10 evaluated balance, mobility or muscle strength. Similarly, 20 of the 32 nervous system outcome measuring tools assessed pain specifically.

**TABLE 5:** Frequency of the 20 most common primary and secondary outcome measuring tools across the 184 included trials, categorised by the method of reporting.

Measuring tools	Primary *n* (%)	Secondary *n* (%)
Participant‐reported		
Numerical rating scale	37 (20.1)	24 (13.0)
Visual analogue scale	37 (20.1)	8 (4.3)
Neuropathy Total Symptom Score	13 (7.1)	14 (7.6)
McGill Pain Questionnaire	11 (6.0)	6 (3.3)
Brief Pain Inventory	7 (3.8)	13 (7.1)
Neuropathic Pain Symptom Inventory	6 (3.3)	19 (10.3)
Daily pain rating scale	6 (3.3)	5 (2.7)
Patient global impression of change	5 (2.7)	31 (16.8)
EuroQoL‐5D	5 (2.7)	13 (7.1)
Short Form‐36	4 (2.2)	11 (6.0)
Traditional Chinese Medicine Symptom Score	4 (2.2)	5 (2.7)
Clinician global impression of change	3 (1.6)	15 (8.2)
Sleep Interference Score	2 (1.1)	14 (7.6)
Short Form‐12	2 (1.1)	6 (3.3)
Assessor‐reported		
Nerve Conduction Study	34 (18.5)	18 (9.8)
Toronto Clinical Scoring System	19 (10.3)	7 (3.8)
Michigan Neuropathy Screening Instrument	16 (8.7)	11 (6.0)
Neuropathy Disability Score	5 (2.7)	9 (4.9)
Berg Balance Scale	5 (2.7)	1 (0.5)

*Note*: *n* equates to the number of included trials, which utilised the specified measuring tool, and the percentage reflects the proportion of the 184 included trials.

### 3.6 Follow‐up durations

**FIGURE 4:**
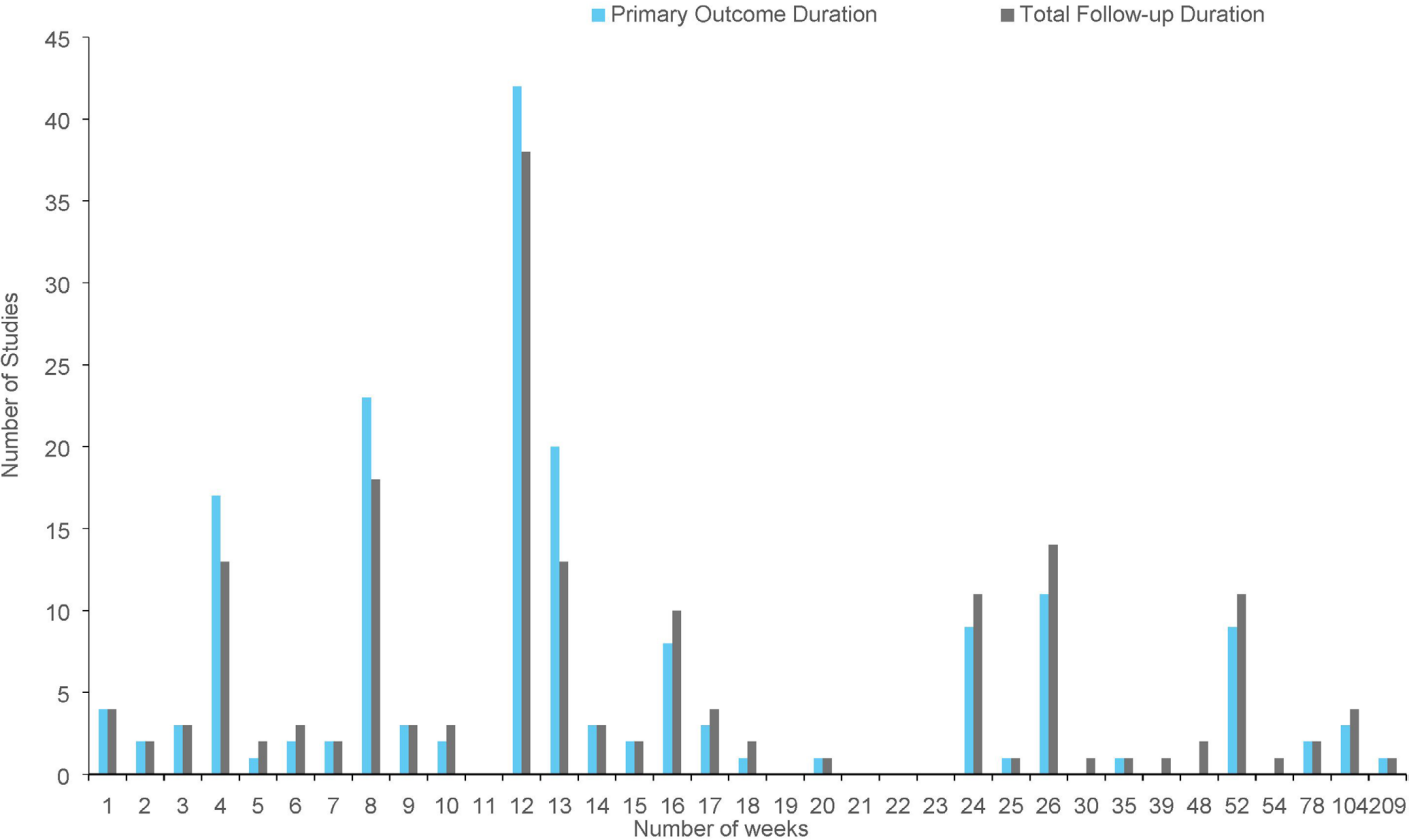
Frequency of primary outcome and total follow‐up durations among included studies, scaled in weeks.

Figure 4 displays the primary outcome and total follow‐up durations of the trials. Of the 184 trials, 8 trials did not specify any outcome durations. The most common primary outcome and total follow‐up durations were similar, with 12 weeks being the most common duration (primary outcome *n* = 42, total follow‐up *n* = 38), followed by 8 weeks (primary outcome *n* = 23, total follow‐up *n* = 18). The longest trial had a primary outcome and total follow‐up duration of 209 weeks (*n* = 1). By contrast, the shortest trials had a primary outcome and total follow‐up duration of 1 week (*n* = 4).

## 4 DISCUSSION

Interventional DSPN trials from the last 5 years have measured a variety of outcomes. The most common outcome was pain, with over two‐thirds of trials measuring it as a primary outcome. Pain was measured in studies conducted across all global regions, suggesting broad recognition of pain as a relevant outcome despite cultural variability. This indicates considerable consistency in recognising the significance of pain in people with DSPN,^5^ with it being the most common symptom and specifically neuropathic pain affecting around one‐third of patients.^28^ However, many trials did not distinguish between neuropathic and nociceptive pain, which may lead to confounding treatment effects.

Adverse events and quality of life were the second and third most common outcomes, respectively, indicating a prioritisation of safety and participants overall wellbeing beyond symptom alleviation. Nervous system outcomes were the most common outcome by taxonomy, with more than 90% of trials measuring this type as a primary outcome. However, the measuring tools used varied across the studies. A total of 32 different measuring tools to measure nervous system outcomes were used, of which 20 specifically measured pain. Similarly, a previous systematic review performed by Mehta et al.,^29^ which investigated pain and physical functioning outcomes in neuropathic pain studies, reported that 24 unique pain intensity measuring tools were used across studies.^29^ Hence, while pain emerged as a primary focus across interventional DSPN trials, the variation in scoring methods underscores the need for a COS recommending standardised tools. To address any cultural variability in the perception of pain, we recommend using standardised, validated tools with cross‐cultural adaptations in future DSPN trials.

The two most frequently used measuring tools for pain were the NRS and VAS, which simply measure pain on a severity index. Self reported questionnaires, such as the Neuropathic Pain Symptom Inventory (NPSI),^30^ have been developed to be specific and differentiate from other non‐neuropathic types of pain. However, these questionnaires were less commonly used across trials. Given the subjective nature of these questionnaires, a combined approach of self and assessor‐reported outcomes may provide a more reliable assessment of DSPN. For example, the Michigan Neuropathy Screening Instrument (MNSI) includes a self reported questionnaire and a clinical examination.^31^ A study investigating the validity of MNSI found that the specificity is more than 90% when the MNSI examination score threshold is set at ≥2.5.^31^ Another measuring tool is the Toronto Clinical Scoring System (TCSS), which also scores DSPN symptoms and assesses clinical signs such as reflexes and sensation.^32^ Unlike the MNSI, the TCSS further stratifies patients based on severity, making it more suitable for longitudinal assessments.^33^

The most common assessor‐reported measuring tool was NCS, which remains the gold standard for the diagnosis of DSPN and serves as a strong predictor for complications such as diabetic foot ulceration and mortality.^3^ NCS measurements are also significantly correlated with clinical diagnostic scores such as the Diabetic Neuropathy Examination (DNE),^32^ reliably reflecting the general clinical status of an individual with DSPN. Despite their advantages, NCS are labour intensive, time‐consuming and expensive. Additionally, determining which nerve and parameter to measure when using NCS to assess DSPN is challenging. This review found that the peroneal motor nerve and sural sensory nerve were the most frequently measured nerves for primary outcomes. In terms of parameter, nerve conduction velocity was the most common primary and secondary outcome. This is consistent with the Rochester Diabetic Neuropathy Study,^34^ which stated that the peroneal motor nerve conduction velocity and sural sensory nerve conduction velocity were the two most sensitive parameters of NCS, with more than a quarter of people with DSPN displaying abnormal readings for these outcomes.^34^ However, NCS are vulnerable to interobserver variability,^25^ which can impact the results of interventional DSPN trials. Therefore, it is recommended to standardise the performance of operators, where possible. Including F‐wave latency in NCS may improve sensitivity, as it detects early nerve dysfunction more precisely than conduction velocities.^35^ Despite its potential, only one study in this review used it,^36^ and it should be more widely adopted. Additionally, since nerve conduction changes take time, trials using NCS as a primary endpoint should last at least 12 weeks to capture treatment effects.

Furthermore, a study comparing NCS and current perception thresholds (CPT) found CPT more effective for assessing DSPN severity.^37^ CPT uses transcutaneous electrical stimulation at different frequencies to target various nerve fibres while NCS only assesses large nerve fibre conduction.^37^ Nevertheless, CPT shows only moderate correlation with clinical or electrophysiological measures and is not a surrogate for histopathological confirmation of axonal degeneration. Thus, CPT is best considered a functional screening tool rather than a direct marker of axonal degeneration.^38^

Considering that small fibre loss induces neuropathic pain,^3^ more emphasis should be placed on investigations evaluating small fibre loss such as CPT, quantitative sensory testing and corneal confocal microscopy (CCM).^3^ Additionally, SUDOSCAN, a relatively novel method of assessing C‐fibre (small fibre) damage in patients with DSPN, could be employed. It involves non‐invasive testing of electrochemical skin conductance, reflecting the sudomotor function of C‐fibres.^39^ Across the trials, these measuring tools were uncommon, but perhaps they should be used in combination with NCS to provide a more precise assessment of DSPN severity, based on both large and small fibre changes.

Assessing physical functioning outcomes is crucial to understand the broader effects DSPN has on participants' daily lives.^40^ Based on the taxonomy by Dodd et al.,^26^ physical functioning was the second most common type of outcome. However, there was a lack of consistency in the unique outcomes measured for physical functioning. After the merging of overlapping verbatim outcomes, 22 unique outcomes categorised as physical functioning were identified. These ranged from sleep interference to balance, muscle strength and walking speed. There were also 35 different measuring tools used, 13 of which measured sleep quality. Similarly, the systematic review by Mehta et al.^29^ found 37 different physical functioning outcome domains,^29^ emphasising the lack of congruence in physical functioning outcome measures.

The primary outcome duration is crucial to determining long‐term efficacy of a novel therapy. A retrospective analysis by Wang et al.^41^ reported that more than half of interventional DSPN trials between 2005 and 2021 had an intervention time of 12 weeks or less,^41^ which is consistent with the findings of this review. To demonstrate treatment response durability, regulatory bodies such as the FDA require at least 12 weeks for interventional trials involving neuropathic pain,^42^ for which almost two‐thirds of trials in this review adhere to, and it was the most common primary outcome duration. Quessy et al.^43^ investigated the magnitude of placebo response for interventional neuropathic pain trials of different durations. The authors found that shorter studies of 4–5 weeks had a higher tendency to exhibit a placebo response compared with longer studies of more than 12 weeks.^43^ Hence, in line with The Neuropathic Pain Special Interest Group's (NeuPSIG's) recommendations,^44^ researchers should endeavour to conduct trials of 12 weeks or more to provide robust efficacy evidence.

Although this systematic review has limitations, careful steps have been taken to overcome or mitigate their impact. Firstly, the search criteria brought about selection bias due to the exclusion of studies published over 5 years ago and studies not in the English language. Outcome saturation was assessed and reached; thus, it is unlikely that studies which could have potentially impacted our findings were missed. To minimise selection bias in other areas, a manual citation search was performed to identify relevant trials which were not indexed in the searched databases, ensuring that the literature search was comprehensive. Moreover, trials at varying stages were included, unlike previous systematic reviews performed by Dovell et al.^19^ and Harman et al.^20^ on outcome reporting for diabetic foot ulceration treatment and type 2 diabetes trials, respectively. In addition to protocols and published trials with results, this systematic review included ongoing trials and completed trials without published results in data extraction. This helps to reduce publication bias as not all trials are published, potentially due to failed treatments or insufficient funding. Although this systematic review was limited to prospective clinical trials, the exclusion of retrospective studies was intended to enhance methodological rigour, given the increased risk of outcome reporting bias associated with retrospective designs.^45^

Next, despite data extraction being primarily performed by only one reviewer, screening and outcome data extraction was completed independently by two reviewers with adjudication from a third, a senior reviewer, reducing the subjectivity of the data extraction process. Lastly, the merging of overlapping outcomes is a subjective process as it is up to the discretion of the reviewer to decide how similar two different outcomes are. To overcome the ambiguity of verbatim outcomes, an established framework published by Young et al.^27^ was used to merge non‐unique verbatim outcomes in a systematic manner.

Although this review found that outcome reporting was relatively consistent, a COS may benefit future interventional DSPN trials by ensuring the inclusion of outcomes that are most relevant and significant to all stakeholders. One potential limitation in developing a COS for DSPN is the dual nature of its manifestations encompassing both positive signs (e.g. pain) and negative signs (e.g. sensory loss or axonal degeneration). Nevertheless, we propose a single, comprehensive COS reflecting the clinical reality that patients often experience both. This holistic, patient‐centred approach supports more relevant and generalisable trial outcomes.

As part of the COMET initiative,^23^ the next step in developing a COS will be a narrative review of qualitative studies involving patients. Qualitative data will complement the quantitative findings from this systematic review, providing a deeper understanding of how DSPN impacts people's lives, including symptoms, functional limitations, psychosocial impacts and which outcomes are most important from their perspective.^46^ Afterwards, focus group interviews with stakeholders will be carried out to explore their priorities and perspectives. International and national opinions will be obtained using surveys disseminated at conferences such as during the annual NEURODiab, which is an international study group for diabetic neuropathy aiming to foster advances in clinical trials.^47^ Lastly, a Delphi process will be undertaken to achieve consensus through a multi‐round anonymous feedback questionnaire.^3^ Qualitative data from the narrative review and focus group interviews will help inform the creation of Delphi surveys by facilitating understanding of why certain outcomes are more important to certain stakeholders and help identify appropriate use of language.^46^ This Delphi process will engage a diverse group of stakeholders, including patients with DSPN, caregivers, healthcare professionals, researchers, charities and industry representatives. The strength of this method is the anonymity and equality of participants, which aids in reducing controlled feedback due to the pressure to conform to a majority group.^48^ After each round of the Delphi process, the collated results will be fed back to participants, and eventually, a consensus will meet among the key stakeholders. This multi‐step process will result in the development of a COS for future DSPN interventional trials, ensuring that patient‐centred and clinically meaningful outcomes are comprehensively identified and incorporated.

Following the development of a COS for DSPN interventional trials, it will be imperative to address the variability in measuring tools. An agreed set of measuring tools would complement the COS and further improve outcome reporting. Prinsen et al.^49^ have published a systematic guideline for the selection of measuring tools. This multi‐stage procedure involves conceptual considerations, a literature search, quality and feasibility analysis, and a consensus agreement.^49^

To summarise, DSPN is a highly prevalent complication of diabetes, carrying a significant burden on individuals^3^ and healthcare systems.^9^ The clinical development of several novel therapies for DSPN may have been hampered by inconsistent outcome reporting.^17^, ^18^ This systematic review provides an overview of outcomes measured in recent DSPN interventional trials, as well as their frequencies and durations. This review highlights the congruence of pain as a primary unique outcome and nervous system outcomes as a primary outcome by taxonomy. However, this review found high variability in measuring tools, especially in assessing nervous systems and physical functioning outcomes. This emphasises the need for the development of a COS for DSPN interventional trials with standardised tools. Future directions following this systematic review include a narrative review of qualitative studies, surveying opinions from key stakeholders and a multi‐round Delphi process. Together, these initiatives will aim to develop a COS for DSPN interventional trials, which may improve outcome reporting, comparability between studies, reduce research waste and publication bias, and create more patient‐centred research. Ultimately, this may provide stronger evidence to guide the development of treatments, healthcare guidelines and policies for DSPN.^21^

## FUNDING INFORMATION

This research did not receive any funding.

## CONFLICT OF INTEREST STATEMENT

Dr. Sasha Smith and Professor Alun Davies have received research grants from Actegy Ltd.

## Supporting information


**Appendix S1.** Search strategy.

**Appendix S2.** Qualtrics (Seattle, United States of America) data extraction form.

**Appendix S3.** Verbatim primary and secondary outcomes classified into unique outcomes.
