# Supplementary material for: A systematic review of outcomes measured in interventional trials in people with diabetic sensorimotor polyneuropathy
Source: Diabet Med. 2025 Sep 12;42(11):e70134. doi: 10.1111/dme.70134 (PMC12535334; doi:10.1111/dme.70134)
Supplement: Supplementary file 1 — Appendix S1. Search strategy. [file DME-42-e70134-s002.docx]

**Appendix 1: Search strategy**

**Ovid MEDLINE® and Embase databases search**

Embase Classic+Embase <1947 to 2023 March 10>

Ovid MEDLINE(R) ALL <1946 to March 10, 2023>

1 (diabet* and (neuropath* or polyneuropath*)).af.

2 (management or treatment* or intervention* or therap*).af.

3 exp Treatment Outcome/ or exp Patient Reported Outcome Measures/

4 outcome*.af.

5 exp Randomized Controlled Trial/ or exp Clinical Trial/

6 (trial* or stud*).af.

7 exp Animals/

8 3 or 4

9 5 or 6

10 1 and 2 and 8 and 9

11 ((diabet* and (neuropath* or polyneuropath*) and (management or treatment* or intervention* or therap*) and (Treatment Outcome or Patient Reported Outcome Measures or outcome*) and (Randomized Controlled Trial or Clinical Trial or (trial* or stud*))) not Animals).af.

12 limit 11 to yr="2018 - 2023

**Cochrane Central Register of Controlled Trials (CENTRAL) database search**

#1 Diabet*

#2 (neuropath* or polyneuropath*)

#3 (management or treatment* or intervention* or therap*)

#4 MeSH descriptor: [Treatment Outcome] explode all trees

#5 MeSH descriptor: [Patient Reported Outcome Measures] explode all trees

#6 outcome*

#7 MeSH descriptor: [Randomized Controlled Trial] explode all trees

#8 MeSH descriptor: [Clinical Trial] explode all trees

#9 (trial* or stud*)

#10 #4 or #5 or #6

#11 #7 or #8 or #9

#12 #1 and #2 and #3 and #10 and #11

With Publication Year from 2018 to 2023, in Trials
