# Supplementary material for: A systematic review of outcomes measured in interventional trials in people with diabetic sensorimotor polyneuropathy
Source: Diabet Med. 2025 Sep 12;42(11):e70134. doi: 10.1111/dme.70134 (PMC12535334; doi:10.1111/dme.70134)
Supplement: Supplementary file 2 — Appendix S2. Qualtrics (Seattle, United States of America) data extraction form. [file DME-42-e70134-s001.pdf]

Imperial College London

Study identity

Title

First Author

DOI

Study style

Study Design

- Randomised Control Trial
- Non-randomised Control Trial

Trial Stage

- Protocol
- Ongoing Trial
- Completed Without Published Results
- Published Paper With Results

Geographical Region

- North America
- South America
- Europe
- Africa
- Asia
- Australia
- International

Intervention Type

- Pharmacological

- ○ Non-pharmacological
- ○ Both

Outcome details

Sample size

Do all patients have diabetic neuropathy?

- ○ Yes
- ○ No

Does the study include patients with Type 1 or Type 2 Diabetes?

- ○ Type 1
- ○ Type 2
- ○ Both
- ○ Not mentioned

Number of centres/clinical trial sites?

Primary Outcomes Measuring Tools (Scoring Systems/questionnaires)

Click to write Choice 1

Click to write Choice 2

Click to write Choice 3

Click to write Form Field 4

Click to write Form Field 5

Primary Verbatim Outcomes i.e. as specified in the study

Click to write Form Field 1

Click to write Form Field 2

Click to write Form Field 3

Click to write Form Field 4

Click to write Form Field 5

Primary Outcomes as per taxonomy

- ☐ Mortality/survival

Physiological/clinical

- ☐ Blood and lymphatic system outcomes
- ☐ Cardiac outcomes
- ☐ Congenital, familial and genetic outcomes
- ☐ Endocrine outcomes
- ☐ Ear and labyrinth outcomes
- ☐ Eye outcomes
- ☐ Gastrointestinal outcomes
- ☐ General outcomes
- ☐ Hepatobiliary outcomes
- ☐ Immune system outcomes
- ☐ Infection and infestation outcomes
- ☐ Injury and poisoning outcomes
- ☐ Metabolism and nutrition outcomes

- ☐ Musculoskeletal and connective tissue outcomes
- ☐ Outcomes relating to neoplasms: benign, malignant and unspecified (including cysts and polyps)
- ☐ Nervous system outcomes
- ☐ Pregnancy, puerperium, and perinatal outcomes
- ☐ Renal and urinary outcomes
- ☐ Reproductive system and breast outcomes
- ☐ Psychiatric outcomes
- ☐ Respiratory, thoracic and mediastinal outcomes
- ☐ Skin and subcutaneous tissue outcomes
- ☐ Vascular outcomes

☐ Perceived health status

☐ Delivery of care, including satisfaction, adherence, withdrawal from treatment

☐ Personal circumstances

☐ Adverse events/effects

☐ Global quality of life

Functioning

- ☐ Physical functioning
- ☐ Social functioning
- ☐ Role functioning
- ☐ Emotional functioning/well-being
- ☐ Cognitive functioning

Resource use

- ☐ Economic
- ☐ Hospital
- ☐ Need for further intervention
- ☐ Societal/carer burden

Secondary Outcomes Measuring Tools (Scoring Systems/questionnaires)

Click to write Choice 1	
Click to write Choice 2	
Click to write Choice 3	
Click to write Form Field 4	
Click to write Form Field 5	
Click to write Form Field 6	
Click to write Form Field 7	
Click to write Form Field 8	
Click to write Form Field 9	
Click to write Form Field 10	

Secondary Verbatim Outcomes i.e. as specified in the study
If more than 10, include the rest in the 10th text box

1

2

3

4

5

6

7

8

9

10

Secondary Outcomes as per taxonomy

- [ ] Mortality/survival

Physiological/clinical

- [ ] Blood and lymphatic system outcomes
- [ ] Cardiac outcomes
- [ ] Congenital, familial and genetic outcomes
- [ ] Endocrine outcomes
- [ ] Ear and labyrinth outcomes
- [ ] Eye outcomes
- [ ] Gastrointestinal outcomes
- [ ] General outcomes
- [ ] Hepatobiliary outcomes
- [ ] Immune system outcomes
- [ ] Infection and infestation outcomes
- [ ] Injury and poisoning outcomes
- [ ] Metabolism and nutrition outcomes
- [ ] Musculoskeletal and connective tissue outcomes
- [ ] Outcomes relating to neoplasms: benign, malignant and unspecified (including cysts and polyps)
- [ ] Nervous system outcomes
- [ ] Pregnancy, puerperium, and perinatal outcomes
- [ ] Renal and urinary outcomes
- [ ] Reproductive system and breast outcomes
- [ ] Psychiatric outcomes

- ☐ Respiratory, thoracic and mediastinal outcomes
- ☐ Skin and subcutaneous tissue outcomes
- ☐ Vascular outcomes

☐ Perceived health status

☐ Delivery of care, including satisfaction, adherence, withdrawal from treatment

☐ Personal circumstances

☐ Adverse events/effects

☐ Global quality of life

Functioning

- ☐ Physical functioning
- ☐ Social functioning
- ☐ Role functioning
- ☐ Emotional functioning/well-being
- ☐ Cognitive functioning

Resource use

- ☐ Economic
- ☐ Hospital
- ☐ Need for further intervention
- ☐ Societal/carer burden

Was the primary outcome and timepoint prespecified in the methods?

○ Primary outcome and timepoint pre-specified

○ Only primary outcome pre-specified

○ Not pre-specified

Primary Outcome duration

Number of follow-ups

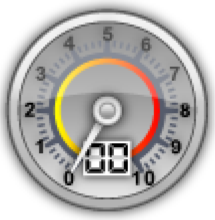

Total follow-up duration

Funder e.g. public/industry

- ◯ Public
- ◯ Industry
- ◯ Not mentioned

Accessibility

Powered by Qualtrics
