# Supplementary material for: A systematic review of outcomes measured in interventional trials in people with diabetic sensorimotor polyneuropathy
Source: Diabet Med. 2025 Sep 12;42(11):e70134. doi: 10.1111/dme.70134 (PMC12535334; doi:10.1111/dme.70134)
Supplement: Supplementary file 3 — Appendix S3. Verbatim primary and secondary outcomes classified into unique outcomes. [file DME-42-e70134-s003.docx]

**Appendix 3: Verbatim primary and secondary outcomes classified into unique outcomes**

| **Unique outcome** | **Verbatim outcomes  (R1)** | **Verbatim outcomes  (R2)** | **Primary**  **(R1)** | **Secondary (R1)** | **Primary (R2)** | **Secondary (R2)** | **Primary (R3; final)** | **Secondary (R3; final)** |
| --- | --- | --- | --- | --- | --- | --- | --- | --- |
| **Self-reported** | | | | | | | | |
| Pain | - Pain - Neuropathic pain - Burning pain - Muscle cramps - Mechanical pain | - Pain - Neuropathic pain - Burning pain - Muscle cramps - Mechanical pain | n=127 | n=64 | n=127 | n=64 | n=127 | n=64 |
| Quality of life | - Quality of life - Self-rated health status | - Quality of life - Self-rated health status - Emotional wellbeing - Emotional functioning - Mental state | n=17 | n=41 | n=23 | n=58 | n=17^1^ | n=41^1^ |
| DSPN-related complaints | - DPN-related complaints - Peripheral neuropathy symptoms - DPN symptoms - Signs and symptoms of DSPN - Neuropathy symptoms | - DPN-related complaints - Peripheral neuropathy symptoms - DPN symptoms - Signs and symptoms of DSPN - Neuropathy symptoms | n=14 | n=8 | n=14 | n=8 | n=14 | n=8 |
| Daily functioning | - Daily life activities - Performance level of routine activities - Self-efficacy behaviours - Self-care activities - Daily functioning | - Daily life activities - Performance level of routine activities - Self-efficacy behaviours - Self-care activities - Daily functioning | n=7 | n=16 | n=7 | n=16 | n=7 | n=16 |
| Patient Global Impression of Change | - Patient Global Impression of Change - Patient Global Impression of Severity | - Patient Global Impression of Change - Patient Global Impression of Severity | n=5 | n=30 | n=5 | n=30 | n=5 | n=30 |
| Anxiety and Depression | - Depression and anxiety - Depressive symptoms - Depression | - Depression and anxiety - Depressive symptoms - Depression | n=5 | n=22 | n=5 | n=22 | n=5 | n=22 |
| **Assessor-reported** | | | | | | | | |
| Nerve conduction velocity | - Nerve conduction velocity | - Nerve conduction velocity | n=41 | n=26 | n=41 | n=26 | n=41 | n=26 |
| Vibration | - Vibration - Vibration perception - Vibration perception threshold - Vibration sensation - Vibration detection | - Vibration - Vibration perception - Vibration perception threshold - Vibration sensation - Vibration detection | n=39 | n=19 | n=39 | n=19 | n=39 | n=19 |
| Numbness | - Numbness | - Numbness | n=38 | n=21 | n=38 | n=21 | n=38 | n=21 |
| Reflexes | - Reflexes | - Reflexes | n=36 | n=21 | n=36 | n=21 | n=36 | n=21 |
| Temperature | - Temperature - Cold detection threshold - Temperature sensitivity - Temperature detection - Thermal sensation - Cold skin sensation - Hot and cold sensation | - Temperature - Cold detection threshold - Temperature sensitivity - Temperature detection - Thermal sensation - Cold skin sensation - Hot and cold sensation | n=36 | n=21 | n=36 | n=21 | n=36 | n=21 |
| Tingling | - Tingling - Paraesthesia - Pricking sensation - Prickling sensation | - Tingling - Paraesthesia - Pricking sensation - Prickling sensation | n=27 | n=13 | n=27 | n=13 | n=27 | n=13 |
| Ataxia | - Ataxia - Unsteadiness | - Imbalance - Balance - Ataxia - Unsteadiness - Postural instability - Functional balance - Static balance | n=15 | n=9 | n=23 | n=16 | n=23^2^ | n=16^2^ |
| Light touch | - Light Touch | - Light Touch | n=21 | n=11 | n=21 | n=11 | n=21 | n=11 |
| Action potential amplitude | - Action potential - Amplitude - Action potential amplitude | - Action potential - Amplitude - Action potential amplitude | n=18 | n=15 | n=18 | n=15 | n=18 | n=15 |
| Blood glucose | - Hba1c - Glycosylated haemoglobin | - Fasting plasma glucose - 1h post-prandial blood glucose - 2h post-prandial glucose - Blood glucose - Fasting blood glucose - Fasting blood sugar levels - Postprandial plasma glucose - Blood sugar - Hba1c - Glycosylated haemoglobin | n=11 | n=22 | n=15 | n=25 | n=15^3^ | n=25^3^ |
| Pinprick | - Pinprick - Pin prick - Pin-prick | - Pinprick - Pin prick - Pin-prick | n=15 | n=9 | n=15 | n=9 | n=15 | n=9 |
| Adverse Events | - Adverse Events | - Adverse Events | n=14 | n=48 | n=14 | n=48 | n=14 | n=48 |
| Foot ulcers/appearance | - Foot ulceration - Feet appearance - Foot deformity - Dry skin - Calluses - Infection - Fissures and ulcers - Hair or nail abnormalities | - Foot ulceration - Feet appearance - Foot deformity - Dry skin - Calluses - Infection - Fissures and ulcers - Hair or nail abnormalities | n=13 | n=9 | n=13 | n=9 | n=13 | n=9 |
| Weakness | - Weakness | - Weakness | n=13 | n=8 | n=13 | n=8 | n=13 | n=8 |

R1: Reviewer 1; R2: Reviewer 2; R3: Reviewer 3.

1. Agreement with R1 as emotional wellbeing and functioning can be classified separately from quality of life.
2. Agreement with R2 as ataxia can include difficulties with balance and postural instability.
3. Agreement with R2 as blood glucose can include fasting glucose, post-prandial glucose, blood sugar, Hba1c and glycosylated haemoglobin.
